# A cross-sectional study of non-suicidal self-injury in adolescent depression: association with demographic characteristics and physiological indicators

**DOI:** 10.3389/fpsyt.2024.1359400

**Published:** 2024-07-25

**Authors:** Yangliuqing He, Yuhan Wei, Yiming Wang, Fenrong Liang, Tianpei Ma

**Affiliations:** ^1^ Clinical Medicine College of Guizhou Medical University, Guiyang, China; ^2^ Department of Psychiatry, Affiliated Hospital of Guizhou Medical University, Guiyang, China

**Keywords:** depression, non-suicidal self-injury (NSSI), adolescents, demographic characteristics, physiological indicators

## Abstract

**Introduction:**

Non-suicidal self-injury (NSSI) is a prevalent concern among adolescents with depression, yet its relationship with demographic characteristics and physiological indicators remains underexplored. This study aimed to investigate these relationships among inpatient adolescents aged 13 to 18 at a hospital affiliated with Guizhou Medical University.

**Methods:**

A cross-sectional study was conducted involving 222 adolescent inpatients diagnosed with depression. Data on NSSI occurrence, demographic variables (gender, only-child status, age), and physiological indicators (ALT, TSH, FT4, PLR, TG, HDLC, LDLC, FT3, NLR, MLR) were collected and analyzed. Statistical analyses, including correlations and group comparisons, were performed to assess the associations between NSSI and these factors.

**Results:**

The prevalence of NSSI among the participants was 40.5%. Significant correlations were found between NSSI and several demographic and physiological factors. Specifically, NSSI was significantly associated with female gender, non-only-child status, younger age, lower ALT levels, higher TSH levels, lower FT4 levels, and higher PLR values. However, no significant differences were observed in TG, HDLC, LDLC, FT3, NLR, or MLR between the NSSI and non-NSSI groups.

**Discussion:**

The findings highlight distinct demographic and physiological profiles associated with NSSI among adolescents with depression. The prevalence rate of NSSI underscores its significance as a behavioral manifestation in this population. Further research should explore the underlying mechanisms linking these factors to better inform targeted interventions and treatment strategies for adolescents experiencing NSSI in the context of depression.

## 1 Introduction

Non-suicidal self-injury (NSSI) refers to the deliberate and repetitive harm to one’s own body without suicidal intent (1). This behavior encompasses various forms such as cutting, scratching, hitting, or burning oneself (2). Adolescence is a peak period for NSSI, especially between the ages of 15 and 17, though the behavior often diminishes in young adulthood (3). Adolescents face rapid physical and psychological changes, as well as adjustments in social roles and expectations, which may lead some to adopt NSSI as a means of coping with stress, regulating emotions, or self-punishment. Consequently, NSSI is particularly prevalent among teenagers and has become a focal point of concern for families, educators, and mental health professionals. Notably, numerous studies have demonstrated a significant association between NSSI and suicidal behaviors and attempts (4).

Globally, the incidence of NSSI exhibits notable variations across different countries and regions, with a general upward trend (5). In some studies, developed countries have reported higher rates of NSSI, possibly linked to their more comprehensive mental health service systems and more open discussions around mental health issues. In contrast, in developing countries, cultural and social factors may contribute to insufficient awareness of mental health issues and limited resources for mental health services, potentially affecting the identification and reporting of NSSI behaviors (6). Therefore, even though high incidence rates of NSSI have been reported in some developing countries, these figures might not fully reflect the actual situation. It is important to note that while various psychiatric disorders can lead to NSSI, depression is the most common associated factor in teenagers.

NSSI is a complex behavior influenced by multiple factors spanning social psychology (6), behaviorism (7), and neurobiology (8), influenced by various factors. In terms of social psychology, NSSI is closely related to an individual’s social environment and psychological state. A supportive social environment positively impacts children’s development, while invalidation, neglect, or abuse may trigger intense negative emotions in children. Depression is a major factor leading to NSSI behavior in adolescents, often associated with adverse family environments and social relationships (9). Research suggests that lack of family support (10), negative parenting styles (11), and distant parent-child relationships (12) significantly increase the risk of adolescent depression and NSSI behavior. Additionally, the resource dilution theory suggests that an increase in the number of children in a family may reduce the resources available to each child, thereby affecting their emotional state and mental health (13), increasing the likelihood of NSSI. Conversely, only children often receive more family support and more positive parenting styles (14), and they tend to have closer relationships with their parents (15). Although some studies suggest that only children may have better mental health outcomes (16, 17), there are also studies with opposing conclusions (18). Furthermore, social psychological factors include social pressures, school environments, and peer relationships, all of which not only influence the onset and development of depression but are also closely related to NSSI behavior. In terms of neurobiology, studies have found associations between NSSI and inflammation, thyroid hormones, and blood lipids.

Previous research indicates that depression patients with NSSI behavior often exhibit higher levels of inflammatory markers, such as C-reactive protein, tumor necrosis factor-alpha, and white blood cells (19). It is believed that the inflammatory response can cause changes in the central nervous system, leading to frontal lobe dysfunction and increased impulsivity, resulting in recurrent NSSI.

However, evidence in this area remains controversial (20–22). In addition, some studies have explored other biomarkers associated with inflammation, such as the neutrophil-to-lymphocyte ratio (NLR), monocyte-to-lymphocyte ratio (MLR), and platelet-to-lymphocyte ratio (PLR), which have been found to significantly change in depression patients and may be associated with NSSI in further studies (23–25). Existing research has found that depression patients often exhibit decreased thyroid function, which is related to dysfunction of the hypothalamic-pituitary-thyroid (HPT) axis (8, 26). Meanwhile, NSSI behavior may also be associated with this endocrine system imbalance, especially affecting emotional regulation and stress response (27, 28). Regarding the relationship between NSSI and blood lipids, some studies have shown a significant correlation between high levels of HDL cholesterol and the occurrence of depression, as well as a strong correlation between high triglyceride levels and impulsive self-harm behavior and suicidal tendencies in young and middle-aged individuals, but there are also studies with completely opposite conclusions. Different studies have inconsistent results regarding the association of total cholesterol, triglycerides, HDL, LDL, and other indicators (29–34).

The choice to focus on NSSI as a specific subgroup for research is motivated by several important reasons. Firstly, NSSI is quite common among adolescents with depression, and research indicates that NSSI is not only closely associated with depression but may also be a precursor to suicidal behavior. Therefore, gaining a deeper understanding of the relationship between NSSI and depression can help prevent and intervene in the serious consequences that these patients may face. Secondly, research suggests that depression patients engaged in NSSI may have different clinical characteristics and physiological indicators than those not engaged in NSSI. By comparing the differences between these two groups of patients, we can better understand the subtypes of depression, providing a basis for personalized diagnosis and treatment. Moreover, studying NSSI also helps to reveal the underlying mechanisms of adolescent mental health issues. Understanding the differences in physiological/neurobiological indicators between depression patients engaged in NSSI and those who are not can provide us with a more comprehensive understanding, helping to develop more effective intervention measures and treatment strategies. In summary, the choice to focus on NSSI as a specific subgroup for research is driven by concern for the group of depression patients and the pursuit of improving personalized treatment outcomes. By delving into the relationship between NSSI and depression, we can better understand the characteristics of this group and provide more scientific guidance for clinical practice.

Through this study, we aim to investigate the demographic and general admission laboratory differences between adolescent depression patients engaged in non-suicidal self-injury (NSSI) and those not engaged in NSSI, to reveal the characteristics and potential mechanisms of different subgroups. The practical significance of this research lies in providing early diagnosis and warning references for NSSI behavior in adolescent depression, and providing more detailed evidence for personalized treatment and intervention strategies, helping depression patients overcome psychological distress and improve their quality of life. By deeply understanding the characteristics of the group of depression patients, we can provide more scientific guidance for clinical practice, promote the development of precision medicine, and contribute to the advancement of the field of mental health.

## 2 Materials and methods

### 2.1 Study participants

A total of 222 adolescents aged 13 to 18 with depression were included from the Department of Psychiatry at Guizhou Medical University Affiliated Hospital. Among them, there were 60 males and 162 females, with a mean age of 15.42 years. Initially, 300 adolescents were enrolled, but due to data loss and some patients dropping out midway, a final cohort of 222 adolescents was included. Data collection for this study occurred from October 2022 to October 2023.

### 2.2 Inclusion and exclusion criteria

Inclusion criteria (1): Participants met the diagnostic criteria for depressive disorders as outlined in the Diagnostic and Statistical Manual of Mental Disorders, 5th Edition (DSM-5), with a HAMD-24 score ≥ 20 (2). According to the DSM-5 definition of Non-Suicidal Self-Injury (NSSI) behaviors, NSSI history was confirmed on the day of admission by two attending psychiatrists or higher-ranking mental health professionals through direct interviews and medical record reviews, with at least one episode of self-injury occurring ≥ 1 time in the past month. Based on the presence or absence of NSSI history, the study participants were categorized into NSSI and non-NSSI groups.

Exclusion criteria (1): Current diagnosis of other mental disorders (2); Presence of severe suicidal behaviors (3); Coexistence of other physical illnesses (4); History of alcohol and substance abuse or addiction.

### 2.3 Methods

#### 2.3.1 Clinical assessment

On the day of admission, a self-designed questionnaire was administered to collect demographic information about the study participants, including gender, age, singleton status, height, weight, and other relevant data. Additionally, trained professionals utilized the 24-item Hamilton Depression Scale (HAMD-24) to assess the severity of depressive symptoms in patients.

#### 2.3.2 Physiological/Neurobiological indicators

All participants underwent fasting venous blood sampling within 24 hours of admission. Electrolytes, lipid profiles, and liver functions were measured using Roche cobas c702; complete blood counts were determined using Sysmex XN-9000; and thyroid function was assessed using Roche cobas e602. Data were obtained from the electronic medical record system. Laboratory results were extracted from electronic medical records to calculate and compare the values of K, Ca, ALT, AST, total protein, TG, TC, HDLC, LDLC, TSH, FT3, FT4, NLR, MLR, and PLR between the NSSI and non-NSSI groups.

### 2.4 Statistical analysis

Data analysis was performed using IBM SPSS Statistics version 26. The Kolmogorov-Smirnov test was applied to assess the normality of data distribution. Normally distributed quantitative data were expressed as mean ± standard deviation (± s), while non-normally distributed quantitative data were presented using median and interquartile range [M (P25, P75)]. Count data were represented as number and percentage (%). Independent sample t-tests, Chi-square tests, or Mann-Whitney U tests were used to compare demographic characteristics and physiological indicators between the NSSI and non-NSSI groups. Independent sample t-tests were utilized for comparisons between groups for normally distributed variables, while Mann-Whitney U tests were used for skewed variables. Binary logistic regression analysis was conducted to identify variables independently associated with NSSI. Odds ratios (OR) and 95% confidence intervals (CI) for independently associated variables were calculated. Model goodness-of-fit was assessed using the Hosmer-Lemeshow test. Receiver operating characteristic (ROC) curve analysis was employed to determine the discriminative thresholds of demographic and physiological indicators for diagnosing NSSI. A two-tailed P < 0.05 was considered statistically significant for all analyses.

## 3 Results

### 3.1 Demographic characteristics of the participants

In this study, 222 adolescents with depression were surveyed (60 males and 162 females), with an average age of 15.42 years. The NSSI group accounted for 40.5% (90/222) of the sample, while the non-NSSI group constituted 59.5% (132/222). Statistically significant differences were observed between the NSSI and non-NSSI groups in terms of gender distribution, age, and Only Child Status (*P* < 0.05). No significant difference in BMI was found between the two groups. The details are shown in **Table 1**.

**Table 1:** Comparison of demographic characteristics between NSSI and non-NSSI groups.

Variables	NSSI Group (n=132)	Non-NSSI Group (n=90)	*X²/t*	*P*
Gender [n (%)]			17.720a	0.000*
Male	22 (16.7)	38 (42.2)		
Female	110 (83.3)	52 (57.8)		
Only Child Status [n (%)]			3.929a	0.047*
Yes	27 (20.5)	29 (32.2)		
No	105 (79.5)	61 (67.8)		
			*Z*	*P*
Age (years)	15 (14,16)	16 (15,17)	-3.718	0.000*
BMI (kg/m²)	19.7 (18.4,22.2)	20.6 (18.7,23.4)	-1.735	0.083

*Indicates statistical significance (P<0.05).

### 3.2 Comparison of blood test results between the study groups

As shown in **Table 2**, significant differences were observed in certain blood parameters between the NSSI group and the non-NSSI group. Specifically:

(1) The level of ALT (Alanine Aminotransferase) was lower in the NSSI group (*P* = 0.025).
(2) The TSH (Thyroid Stimulating Hormone) level was higher in the NSSI group (*P* = 0.011).
(3) The FT4 (Free Thyroxine) level was lower in the NSSI group (*P* = 0.007).
(4) The PLR (Platelet to Lymphocyte Ratio) was higher in the NSSI group (*P* = 0.039).

**Table 2:** Comparison of blood test results between NSSI and non-NSSI groups.

Variables	NSSI Group (n=132)	Non-NSSI Group (n=90)	*Z*	*P*
Ca	2.28 (2.24,2.36)	2.23 (2.25, 2.33)	-0.458	0.647
ALT	10.4 (7.33,14.65)	11.7 (8.75,19.25)	-2.239	0.025*
AST	16.25 (13.65,19.33)	15.6 (13.28,18.75)	-0.599	0.549
TG	0.91 (0.78,1.28)	0.92 (0.71,1.22)	-0.69	0.49
HDLC	1.20 (1.07,1.42)	1.18 (1,1.39)	-1.361	0.173
TSH	1.84 (1.19,2.85)	1.49 (1.01,2.14)	-2.53	0.011*
FT4	15.39 (14.06,17.13)	16.61 (14.76,17.82)	-2.68	0.007*
WBC	5.75 (5.02,6.70)	5.99 (5.08,6.75)	-0.766	0.444
NLR	1.23 (0.91,1.63)	1.26 (1.02,1.58)	-0.647	0.518
MLR	0.19 (0.15,0.25)	0.20 (0.15,0.24)	-0.22	0.826
PLR	115.10 (88.83,141.31)	104.80 (80.76,126.57)	-2.069	0.039*
			*X²/t*	*P*
K	4.24 ± 0.30	4.20 ± 0.30	-1.131	0.259
LDLC	2.13 ± 0.57	2.12 ± 0.57	-0.106	0.916
Total Protein	67.65 ± 5.13	66.80 ± 5.49	-1.181	0.239
TC	3.72 ± 0.69	3.63 ± 0.71	-0.925	0.356
FT3	5.06 ± 0.66	5.20 ± 0.78	1.428	0.155

*Indicates statistical significance (P<0.05).

No significant differences were found between the two groups regarding Ca (Calcium), K (Potassium), AST (Aspartate Aminotransferase), TG (Triglycerides), HDLC (High-Density Lipoprotein Cholesterol), LDLC (Low-Density Lipoprotein Cholesterol), TC (Total Cholesterol), total protein, WBC (White Blood Cell Count), NLR (Neutrophil to Lymphocyte Ratio), MLR (Monocyte to Lymphocyte Ratio), and FT3 (Free Triiodothyronine).

As shown in **Table 3**, the binary logistic regression analysis indicated that gender (male vs. female, *OR*=0.274, *P*<0.001), singleton status (no vs. yes, *OR*=1.849, *P*=0.049), thyroid-stimulating hormone (TSH) level (*OR*=1.253, *P*=0.037), free thyroxine (FT4) level (*OR*=0.89, *P*=0.043), age (*OR*=0.716 per year increase, *P*<0.001), and platelet-to-lymphocyte ratio (PLR) (*OR*=1, *P*=0.047) were significant independent risk factors for the occurrence of non-suicidal self-injury (NSSI) in adolescents with depression. To assess the goodness of fit of the model, the Hosmer-Lemeshow test was utilized in this study. The test results revealed a Chi-square value of 8.247 with 8 degrees of freedom and a p-value of 0.409, indicating no significant difference between the predicted and observed data at the 5% significance level. Thus, this demonstrates that the model has a good fit. Consequently, the logistic regression model employed in this study is suitable for analyzing the data and can effectively predict and explain the relevant factors associated with the occurrence of NSSI in adolescents with depression.

**Table 3:** Binary logistic regression model analysis of demographic and physiological indicators in relation to NSSI.

Variables	OR (95% CI)	*P*
Gender (1)	0.274	<0.001*
Only Child Status	1.849	0.049*
TSH	1.253	0.037*
FT4	0.89	0.043*
Age	0.716	<0.001*
PLR	1	0.047*

*Indicates statistical significance (P<0.05).

**Figure 1:**
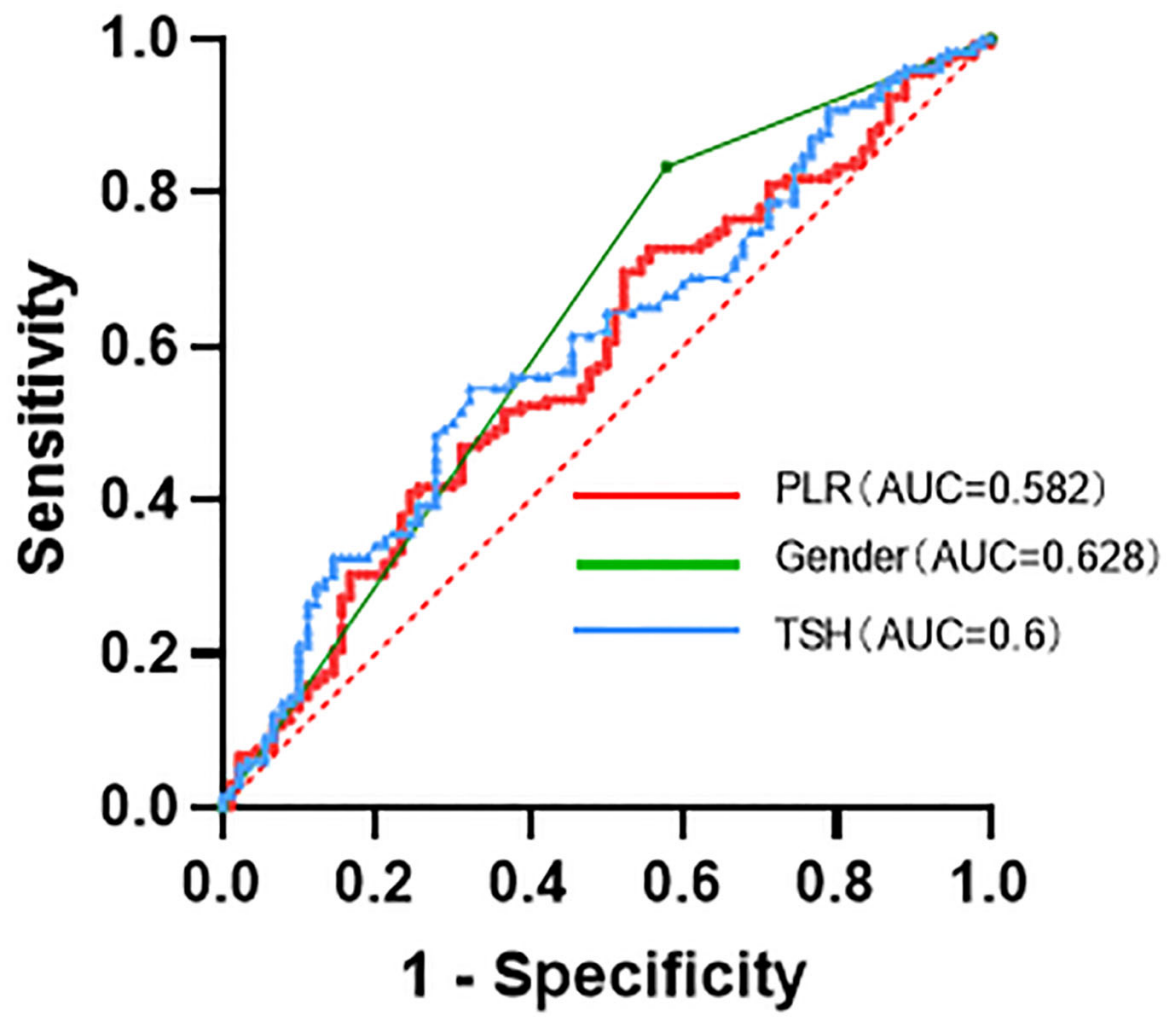
Characteristics of the ROC (receiver operating characteristic) curves for TSH, PLR, and gender in discriminating NSSI from non-NSSI subjects.

As shown in **Table 4** and **Figure 1** the ROC curves for TSH, PLR, and gender are represented in blue, red, and green lines, respectively. The AUC for TSH is 60.0%, with a sensitivity of 54.5% and specificity of 45.5%. PLR is shown in red with an AUC of 58.2%, a sensitivity of 69.7%, and a specificity of 30.3%. Gender is represented by a green line, with an AUC of 62.8%, a sensitivity of 83.3%, and specificity of 42.2%.

**Table 4:** Characteristics of the ROC (receiver operating characteristic) curves for TSH, PLR, and gender in discriminating NSSI from non-NSSI subjects.

Variables	AUC	OR (95% CI)	Cut-off	sensitivity	specificity	*P*
TSH	0.6	0.525–0.675	1.785	54.5%	45.5%	0.011*
PLR	0.582	0.506–0.658	96.853	69.7%	30.3%	0.039*
Gender	0.628	0.551–0.704	–	83.3%	42.2%	0.001*

*Indicates statistical significance (P≤0.05).

The '-' in the Gender row of **Table 4** signifies that no specific cut-off value was applicable for this variable.

## 4 Discussion

This study extensively examines the relationships among demographic characteristics, physiological indicators, and self-injurious behaviors in adolescents with depression. The results suggest that female adolescents and those who are not only children are more prone to self-injurious behaviors. Compared to adolescents without NSSI, those with NSSI tend to be younger and have lower levels of ALT and FT4 but higher levels of TSH and PLR. Notably, other variables such as TG, HDLC, LDLC, FT3, NLR, and MLR did not show significant differences between groups.

### 4.1 The relationship between family structure and NSSI in adolescents with depression

Previous studies have indicated that only children might have better mental health outcomes (16, 17), but conflicting views exist (18). Our study finds that in adolescents with depression, only children are less likely to engage in self-harm compared to those with siblings. This could be related to higher family support received during childhood. Immediate and positive responses to various needs in childhood might lead only children to develop a more positive cognitive pattern, viewing stress as a positive influence (35). Additionally, the resource dilution theory suggests that only children receive more family support, positive parenting (14), and closer parent-child relationships (15), thereby reducing the likelihood of resorting to self-harm to alleviate negative emotions during depressive episodes.

### 4.2 The relationship between gender and NSSI in adolescents with depression

Although some studies indicate differences in the types of self-harm behaviors between males and females (36), with females more likely to seek medical help (37), overall, self-harm is more prevalent among females. Female adolescents are more inclined to regulate emotions and exert self-control through cutting and scratching, while male adolescents may prefer hitting, punching, and burning (38). This might explain the contradictory prevalence rates in studies with unclear definitions of self-harm. The gender difference could stem from females being more emotionally sensitive and intricate, making them more susceptible to psychological issues. Unlike males, who may seek the thrill of impulsivity, females are more inclined to use self-harm for emotional regulation and self-control, explaining why males might have more alternative behaviors for self-injury (39).

### 4.3 The relationship between age and NSSI in adolescents with depression

Our study observed that the age of the NSSI group [15 (14, 16)] is slightly lower than the non-NSSI group [16 (15, 17)], consistent with past research indicating that NSSI behavior peaks in early adolescence (around 15 years old (3)) and then declines (40, 41). This trend may be linked to the immaturity of the frontal cortex in early adolescence, coupled with physiological and psychological changes. As adolescents mature into late adolescence and early adulthood, their emotional control, facilitated by the development of the frontal cortex, becomes more refined, thereby reducing the incidence of self-harm (40, 42).

### 4.4 The relationship between physiological/neurobiological indicators and NSSI in adolescents with depression

#### 4.4.1 ALT (Alanine Aminotransferase)

In our investigation of adolescents with depression, those engaging in non-suicidal self-injury (NSSI) behaviors exhibited lower ALT levels compared to their counterparts without NSSI. While existing literature does not explicitly establish a direct connection between ALT fluctuations and depression, and there is a scarcity of research specifically addressing the relationship between ALT and NSSI, previous studies have hinted at a link between non-alcoholic fatty liver disease (NAFLD) and depression, albeit with unclear underlying mechanisms (43). Potential mechanisms may include cytokine-mediated inflammation (44, 45), activation of the hypothalamic-pituitary-adrenal(HPA) axis, and the impact of insulin resistance on neurotransmission. Notably, despite prior research indicating possible elevations in ALT in the context of depression, our study, focusing on a subtype of depression, namely adolescents with NSSI, reveals relatively lower ALT levels. This may be associated with metabolic, inflammatory, and insulin resistance factors related to depression, yet a more in-depth investigation is required for a conclusive explanation.

#### 4.4.2 FT4 (free thyroxine) and TSH (thyroid-stimulating hormone)

In our study of adolescents with depression, those engaging in non-suicidal self-injury (NSSI) behaviors demonstrated lower FT4 levels and higher TSH levels. This aligns with previous research establishing a connection between depression and thyroid dysfunction (46). Notably, a large-scale study reported a significant reduction in thyroid hormone levels in depression patients (47). Exploring the elevation of TSH, the TRH (Thyrotropin-Releasing Hormone) hypothesis of depression posits that decreased 5-HT function leads to increased TRH secretion, maintaining normal thyroid hormone levels (48, 49). Additionally, studies have shown reductions in FT4 levels and nocturnal TSH responses in individuals with a history of suicidal behavior and depression (27). This suggests a potential role of central TRH functional deficiency in the pathogenesis of suicidal behaviors. Further research could illuminate the significant role of central TRH in NSSI behaviors among adolescents with depression.

#### 4.4.3 PLR (Platelet-to-lymphocyte ratio)

The study found that in adolescent patients with depression, those with NSSI (non-suicidal self-injury) behaviors exhibited higher PLR levels. Previous research has highlighted that somatic inflammatory diseases and increased pro-inflammatory markers are associated with a higher risk of depression (50, 51), indicating that inflammation may play a significant role in the onset and progression of depression. NLR (Neutrophil-to-Lymphocyte Ratio), PLR, and MLR (Monocyte-to-Lymphocyte Ratio) are inflammatory markers calculated from complete blood cell counts and are easily accessible and highly sensitive. Thus, the relationship between these markers and depression has been of considerable interest (52, 53). To our knowledge, prior to our study, only one study conducted in China explored the relationship between NLR, PLR, MLR, and NSSI. That study found that adolescents with mood disorders and NSSI behaviors had significantly elevated levels of MLR and PLR. However, in our research, we found a significant increase in PLR in adolescents with NSSI and depression, while MLR and NLR did not show significant differences. This suggests that PLR might be more indicative than MLR and NLR in assessing self-harming behaviors in adolescents. The increase in PLR among adolescents with depression and NSSI behavior could be linked to its impact on peripheral inflammation and serotonin neurotransmission. Previous research indicates that NSSI behavior is stress-related, which might increase platelet counts through activation of the sympathetic nervous system (54) Increased platelets can induce changes in endothelial permeability, leading to higher levels of peripheral inflammation (55). Additionally, platelets are rich in serotonin, which is involved in the production, reuptake, and metabolism of serotonin (56, 57). Studies have found that aggression and impulsivity in suicide attempters are associated with serotonin content in platelets and the total amount of 5-HT.

Overall, this study delved into the intricate relationship between various characteristics of adolescents with depression and non-suicidal self-injury (NSSI) behaviors. Factors such as gender, family structure (being an only child), age, thyroid function, liver function, and inflammation levels may play pivotal roles in self-injurious behaviors. Our findings further corroborate the independent influence of gender, being an only child, TSH, FT4, age, and PLR on the occurrence of NSSI in individuals with depression. When discussing our research findings, it’s important to note the significant discrepancies in NSSI rates across different countries and regions. Developed countries typically report higher rates of NSSI occurrence, possibly due to their more comprehensive mental health service systems and a more open discourse surrounding mental health issues. In contrast, developing countries may have insufficient awareness of mental health issues due to cultural and societal influences, as well as limited mental health service resources, which could affect the identification and reporting of NSSI behaviors. Therefore, our research findings may be influenced by these inter-country differences, particularly considering our study was conducted in China. Future research could delve deeper into the variations in NSSI behaviors among different countries and regions, taking into account the influence of sociocultural factors on these differences. Additionally, our findings hold significant social and cultural implications. By identifying risk factors for NSSI behaviors in adolescents with depression, we can offer valuable adjunct tools to aid clinicians in better assessing patients’ risk levels. However, it’s crucial to recognize that using these diagnostic adjunct tools in practice may raise ethical considerations and potential harm. Thus, we recommend adopting a cautious approach when utilizing these diagnostic adjunct tools, integrating clinical judgment, and considering other potential influencing factors to avoid infringing upon patient privacy and making overly stigmatizing or judgmental assessments. Lastly, we acknowledge several limitations in this study, including sample limitations, unaccounted potential confounding factors, biases in data collection and measurement methods, and limitations in statistical analysis. Future research should aim to validate these limitations further and gain a better understanding of adolescent depression and its associated mechanisms of non-suicidal self-injury to develop more effective intervention and treatment strategies.

## 5 Conclusion

The findings of this study provide important insights into our understanding of adolescent depression and self-harming behaviors. Factors such as gender, being an only child, thyroid function, liver function, and inflammation levels may play pivotal roles in NSSI behaviors among adolescents with depression, necessitating further research to delve deeper into these factors and validate them. Through ROC curve analysis, we found that TSH, PLR, and gender have potential diagnostic value in distinguishing between adolescent depression cases with and without NSSI behaviors. These indicators may serve as adjunct tools to aid in identifying the risk of NSSI behaviors among adolescents with depression in subsequent clinical practice.

## Data availability statement

The raw data cannot be shared at this time as the data also forms part of an ongoing study. Requests to access the datasets should be directed to contact the corresponding author. Requests to access these datasets should be directed to 438124323@qq.com.

## Ethics statement

The studies involving humans were approved by Guizhou Medical University Affiliated Hospital Medical Ethics Committee. The studies were conducted in accordance with the local legislation and institutional requirements. Written informed consent for participation in this study was provided by the participants’ legal guardians/next of kin.

## Author contributions

YH: Conceptualization, Data curation, Formal analysis, Investigation, Methodology, Project administration, Software, Supervision, Validation, Visualization, Writing – original draft, Writing – review & editing. YHW: Methodology, Writing & review & editing. YMW: Writing – review & editing, Methodology, Project administration, Software, Supervision. FL: Data curation, Writing & review & editing. TM: Resources, Writing & review & editing.
